# Supplementary material for: Genome-encoded ABCF factors implicated in intrinsic antibiotic resistance in Gram-positive bacteria: VmlR2, Ard1 and CplR
Source: Nucleic Acids Res. 2023 Mar 23;51(9):4536–54. doi: 10.1093/nar/gkad193 (PMC10201436; doi:10.1093/nar/gkad193)
Supplement: gkad193_Supplemental_Files [file gkad193_supplemental_files.zip › Obana_Supplementary_Information.pdf]

SUPPLEMENTARY ONLINE MATERIALS

for

Genome-encoded ABCF factors implicated in intrinsic antibiotic resistance in Gram-positive bacteria: VmlR2, Ard1 and CplR

Nozomu Obana[1,2,*,#], Hiraku Takada[3,4,#], Caillan Crowe-McAuliffe[5,#], Mizuki Iwamoto[6], Artyom A. Egorov[4], Kelvin J.Y. Wu[7], Shinobu Chiba[3,8], Victoriia Murina[9], Helge Paternoga[5], Ben I.C. Tresco[7], Nobuhiko Nomura[2,6], Andrew G. Myers[7], Gemma C. Atkinson[4], Daniel N. Wilson[5,*], Vasili Hauryliuk [4,10,11,*]

[1] Transborder Medical Research Center, Faculty of Medicine, University of Tsukuba, Tsukuba, Japan

[2] Microbiology Research Center for Sustainability (MiCS), University of Tsukuba, Tsukuba, Japan

[3] Faculty of Life Sciences, Kyoto Sangyo University, Motoyama, Kamigamo, Kita-Ku, Kyoto, 603-8555, Japan

[4] Department of Experimental Medical Science, Lund University, Lund, Sweden

[5] Institute for Biochemistry and Molecular Biology, University of Hamburg, Martin-Luther-King-Platz 6, 20146 Hamburg, Germany

[6] Faculty of Life and Environmental Sciences, University of Tsukuba, Tsukuba, Japan

[7] Department of Chemistry and Chemical Biology, Harvard University, Cambridge, MA, USA

[8] Institute for Protein Dynamics, Kyoto Sangyo University, Japan

[9] Department of Molecular Biology, Umeå University, Umeå, Sweden

[10] University of Tartu, Institute of Technology, Tartu, Estonia

[11] Science for Life Laboratory, Lund, Sweden

[#] designates equal contribution

* to whom correspondence should be addressed:

Nozomu Obana, obana.nozomu.gb@u.tsukuba.ac.jp

Daniel N. Wilson, daniel.wilson@chemie.uni-hamburg.de

Vasili Hauryliuk, vasili.hauryliuk@med.lu.se

Supplementary Table 1. Strains, plasmids and oligonucleotides used in this study.

The table is provided as a separate excel file. Details of plasmid and stain construction are also provided in the **Supplementary Table 1**. *B. subtilis* strains used in the study were trpC2 Δ*vmlR* (1).

Supplementary Table 2. Cryo-EM data collection, modelling and refinement statistics.

**Data collection and processing**	
Nominal magnification (×)	165 000
Acceleration voltage (kV)	300
Spherical aberration	2.7
Energy filter slit (eV)	20
Pixel Size (Å)	0.82
Defocus range (μm)	-0.7 to -1.9
Electron flux ($e^-/Å^2/s$)	7.13
Electron fluence ($e^-/Å^2$)	28.52
Exposure time (s)	4
Number of frames	20
Number of micrographs	6 384
Number of particles	140 804
Map resolution (masked, Å)	2.9
**Model**	
Number of atoms	144 765
RNA residues	4 584
Protein residues	5 859
**RMS deviations**	
Bond length (Å)	0.004
Angles (°)	0.664
**Validation**	
MolProbity score	1.40
Clash score	4.27
Map-model FSC at 0.5 (Å)	2.97
Map-model cross-correlation (masked volume)	0.87
Rotamer outliers (%)	0.97
Cβ outliers	0.00
**Ramachandran statistics (%)**	
Outliers	0.07
Allowed	3.05
Favoured	96.88
Z-score	-1.03

Supplementary Table 3. Multiple sequence alignments of ARE-ABCF leader peptides.

The table is provided as a separate excel file. Complete multiple sequence alignments of leader peptides that regulate expression of CplR, VmlR, VmlR2, and LsaA generated by uORF4u (2).

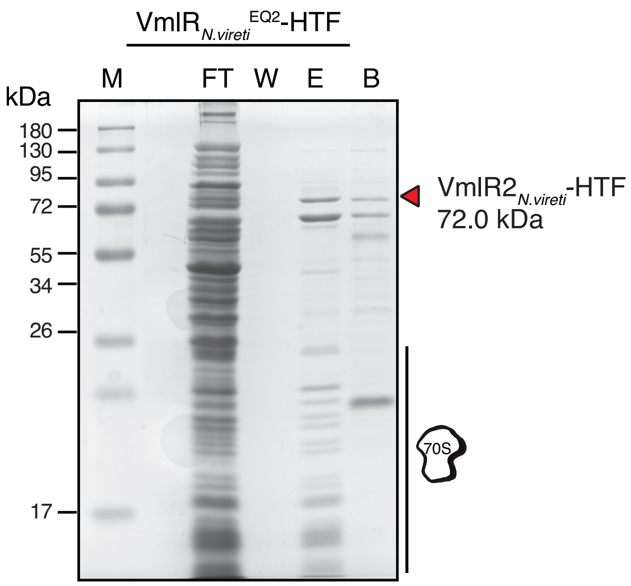

Supplementary Figure 1. Preparation of samples for cryo-EM reconstructions.

Affinity purification of C-terminally HTF-tagged VmlR2$_{N.vireti}^{EQ2}$ expressed in *B. subtilis* VHB220 (Δ*vmlR thrC*::P$_{hy\text{-}spnak}$ -vmlR2$_{N.vireti}^{EQ2}$-*HTF*; HTF stands for His$_6$-TEV-FLAG$_3$). Samples: marker: 2 µL of molecular weight marker; flowthrough: 10 µL; wash: 10 µL of last wash before specific elution; elution: 10 µL of elution with FLAG$_3$; beads: 2 µL of 2xSDS loading buffer boiled with 50 µL of beads. The samples were resolved on 12% SDS-PAGE gel.

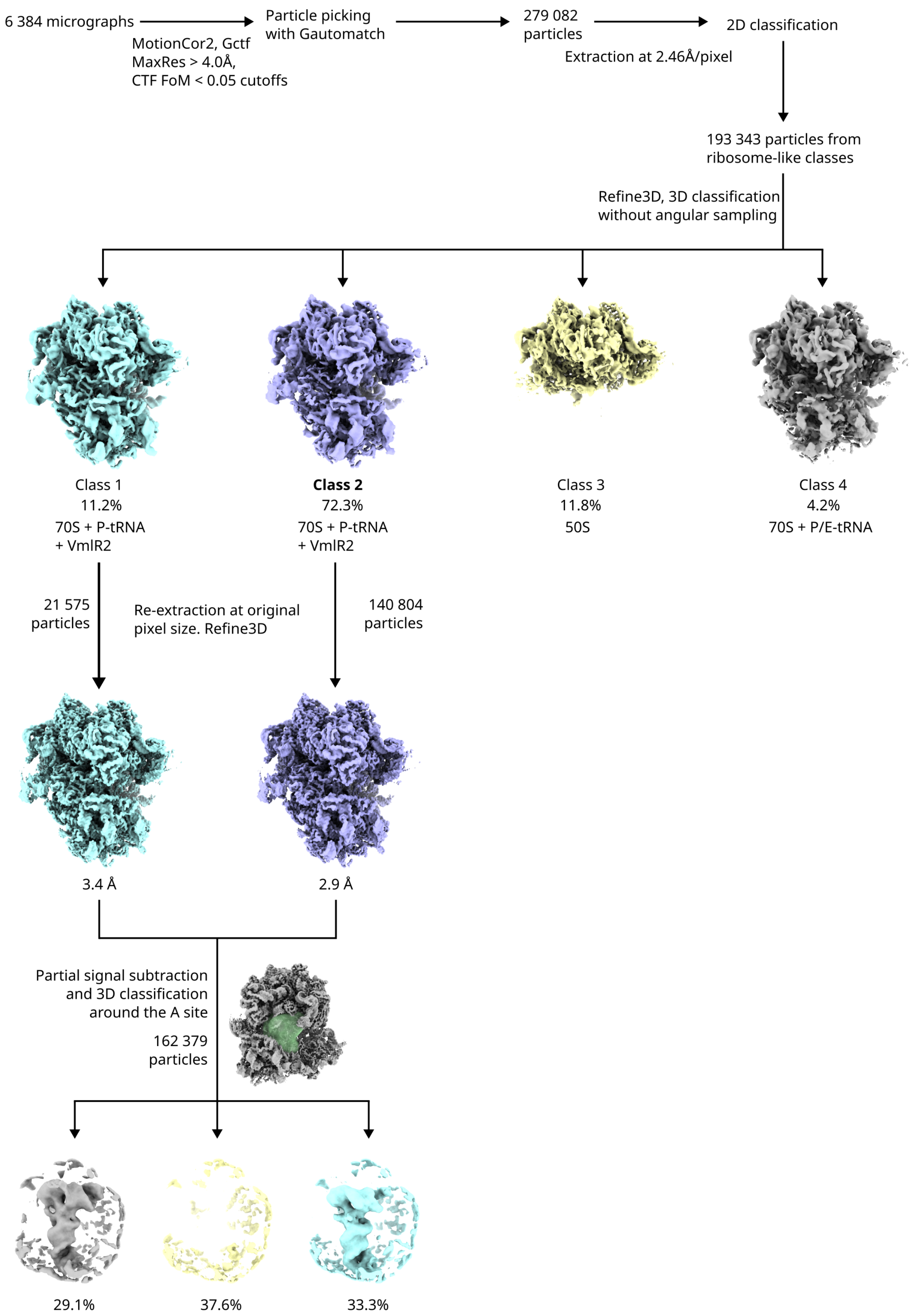

Supplementary Figure 2. Cryo-EM processing scheme for VmlR2-70S.

See also methods for details.

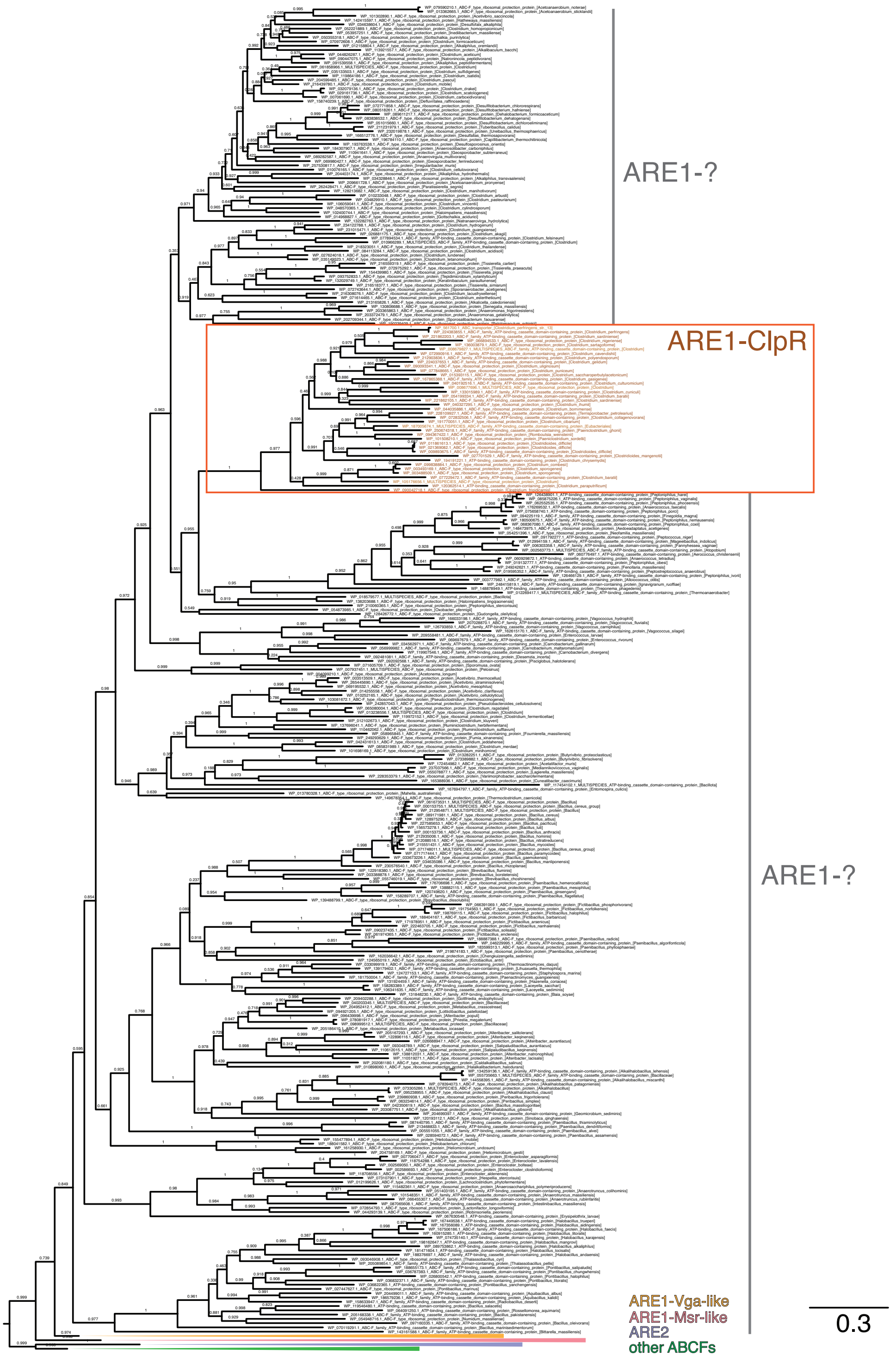

Supplementary Figure 3. Phylogenetic distribution of CplR. Sequences were retrieved from the NCBI RefSeq-Select database using BlastP.

The core CplR group is found in various clostridia genera: *Terrisporobacter*, *Clostridium*, *Clostridoides* and *Paeniclostridia*. However, there is a large diversity of CplR close relatives in bacillota, for which we can not rule out orthology with CplR. Numbers on branches are FastTree bootstrap support values on a scale of 0-1. Branch lengths are proportional to the number of amino acid substitutions as per the scale bar.

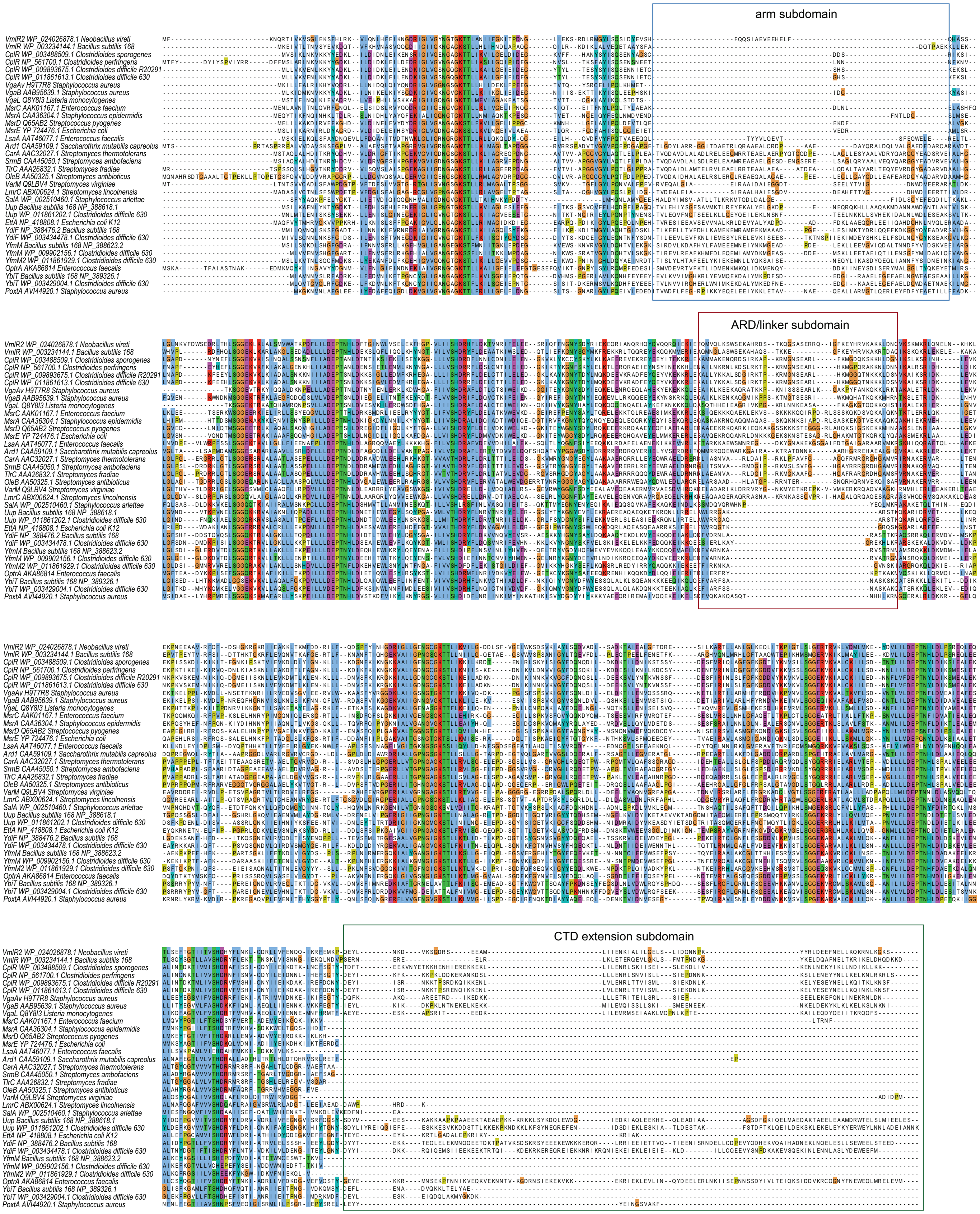

Supplementary Figure 4. Multiple sequence alignment of selected ABCF proteins.

Boxes show the three variable subdomains: the arm, ARD (also referred to as linker), which separates the two conserved ABC domains, and C-terminal domain (CTD).

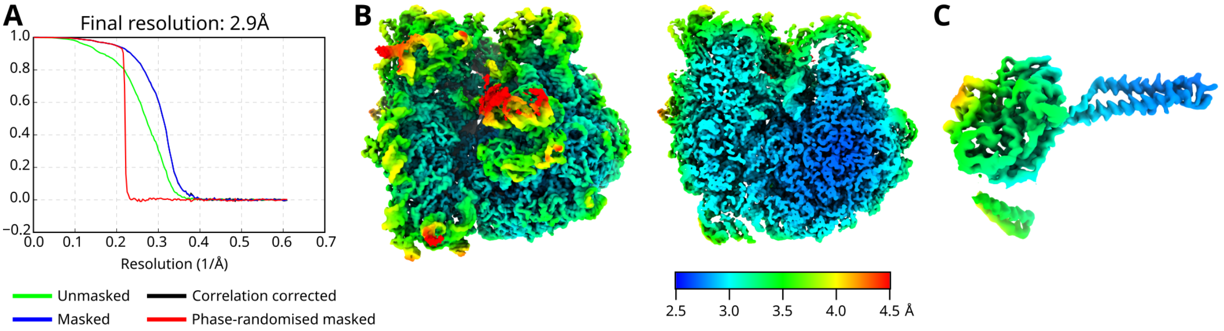

Supplementary Figure 5. FSC curves and local resolution of the VmlR2-EQ$_2$-70S volume.

(**A**) FSC curves. (**B**) Whole volume (left) and cut-through (right) coloured according to local resolution. (**C**) Isolated density for VmlR2 coloured according to local resolution. Non-sharpened maps are shown.

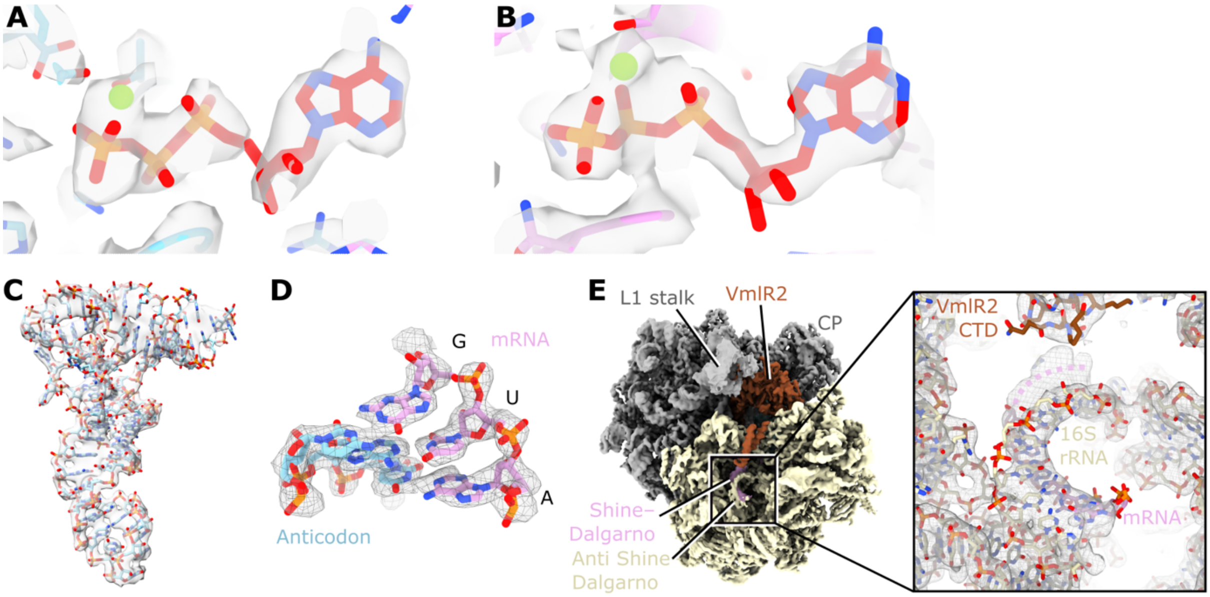

Supplementary Figure 6. Density features of the VmlR2–70S complex.

(**A**) Density and model corresponding to the peripheral NTP-binding site, looking towards NBD2. A putative Mg^{2+} ion is sown as a green sphere. Density if from a post-processed map sharpened with Guinier-estimated B factor. (**B**) Same as **A**, but for the interior NTP-binding site, looking towards NBD1. (**C**) Isolated density and model of the distorted P-tRNA. The modelled residues correspond to an fMet-initiator tRNA. The CCA-3′ end is poorly resolved and not included in the model. (**D**) Isolated density and model for the codon-anticodon interaction. The codon is modelled as AUG (pink). The anticodon is shown in blue. (**E**) View of the VmlR2-70S complex focusing on the mRNA exit channel. Density corresponding to a putative Shine-Dalgarno—anti-Shine-Dalgarno interaction is indicated.

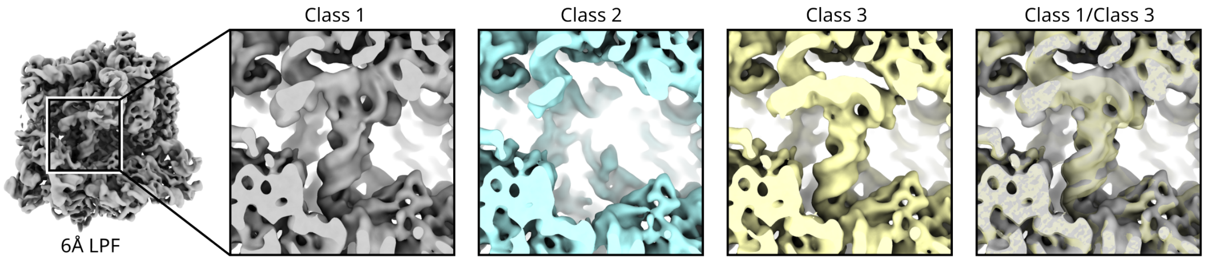

Supplementary Figure 7. A substoichiometric tRNA occupies the A site of the VmlR2-70S complex. View of three sub-classified volumes (see **Supplementary Figure 3**) focusing on the A site. Volumes were low-pass-filtered to 6 Å. Inset shows A site with tRNA (classes 1 and 3) or without ligand (class 2). The right-most panel shows class 1 with transparent density for class 3 overlaid, revealing a different A-tRNA conformation.

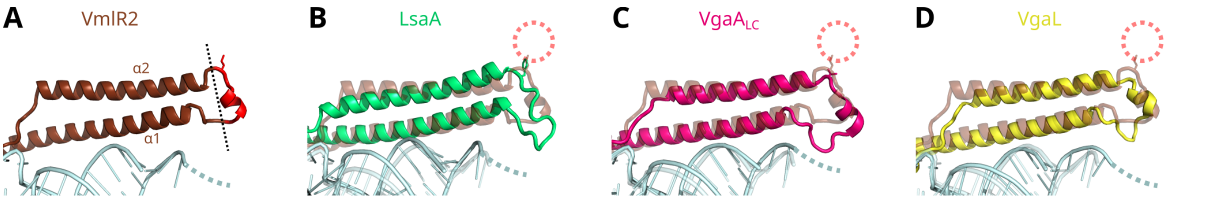

Supplementary Figure 8. Comparison of interdomain linkers between selected ARE-ABCFs.

(**A**) The VmlR2 interdomain linker (brown) with distorted P-tRNA (cyan). Dashed cyan lines indicate the approximate path of the tRNA CCA-3′ end, which was not modelled. Residues which are inserted compared to *B. subtilis* VmlR (see **Figure 1B**) are shown in red and demarcated by the black dotted line. Ile261, which reaches into the PTC, is shown as a stick representation. (**B**) Comparison of the LsaA (PDB ID 7NHK) and VmlR2 interdomain linkers. The red dashed circle indicates the approximate site of PLS_A binding. (**C**) As for **B** but with $VgaA_{LC}$ (PDB 7NHL). (**D**) As for B but with VgaL (PDB ID 7NHN). Models were aligned by 23S rRNA.

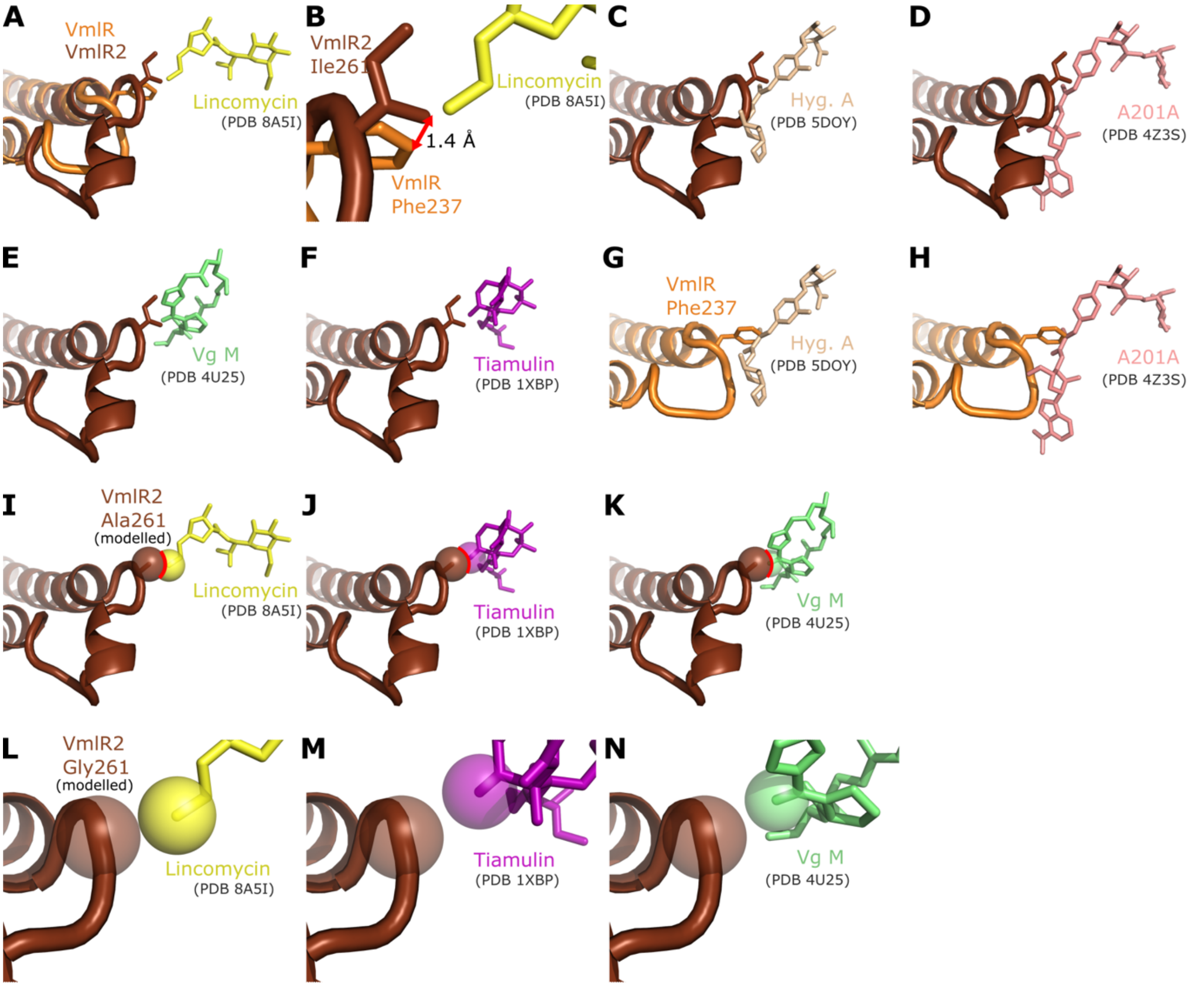

Supplementary Figure 9. Overlap between ABCF-ARDs and selected antibiotics.

(**A**) The VmlR2 (brown) and *B. subtilis* VmlR (orange) ARDs are similarly positioned with respect to the PLS_A binding site. For reference, lincomycin (PDB ID 8A5I) has been superimposed. (**B**) Close view of A, showing a 1.4 Å distance between the most-distal atoms of the VmlR2 and the *B. subtilis* VmlR ARDs. (**C**, **D**) Extensive predicted overlaps between the VmlR2 ARD and hygromycin A (**C**, beige, PDB ID 5DOY) and A201A (**D**, salmon, PDB ID 4Z3S) (3). (**E**, **F**) Same view as in **A** but with virginiamycin M (**E**, green, PDB ID 4U25) or tiamulin (**F**, purple, PDB ID 1XBP) superimposed (4). (**G**, **H**) As for **C** and **D** but with the *B. subtilis* VmlR ARD still showing extensive overlaps. (**I-K**) Predicted overlaps between the VmlR2 I261A variant and lincomycin (**I**), tiamulin (**J**), and virginiamycin M (**K**) (5). (**L-N**) As for panels I-K but for a predicted VmlR2 I261G variant. The Gly261 α-carbon is represented as a sphere. A closer view is shown for clarity. Transparent spheres represent van der Waals radii; red lines indicate clashes. Models were aligned by 23S rRNA.

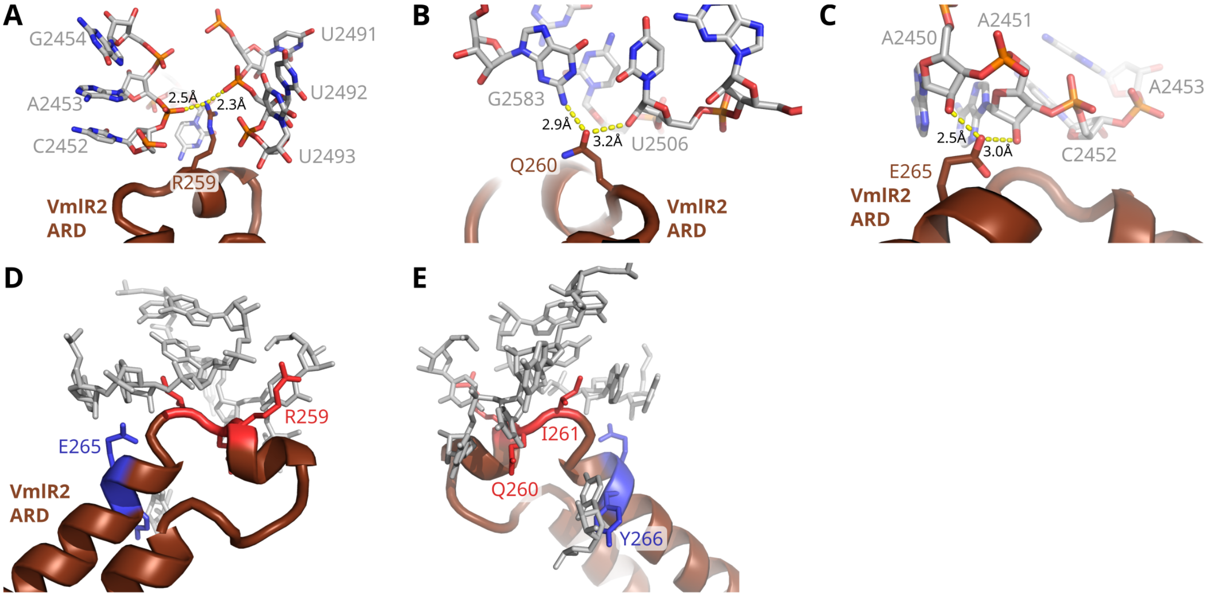

Supplementary Figure 10. Interactions between the VmlR2 ARD and the 23S rRNA.

(**A**) VmlR2 (brown) Arg259 forms H-bonds with 23S rRNA (grey) phosphate groups. (**B**) VmlR2 Gln260 interacts with G2583 and U2506 (**C**) VmlR2 E265 is within hydrogen-bonding distance of 23S rRNA A2450 and A2451. (**D**) Overview of alanine mutants tested in the VmlR2 ARD. Residues that, when substituted with alanine, substantially shifted the antibiotic resistance profile are shown in blue. Residues for which an alanine substitution had no effect are shown in red. (**E**) same as D but with a different view.

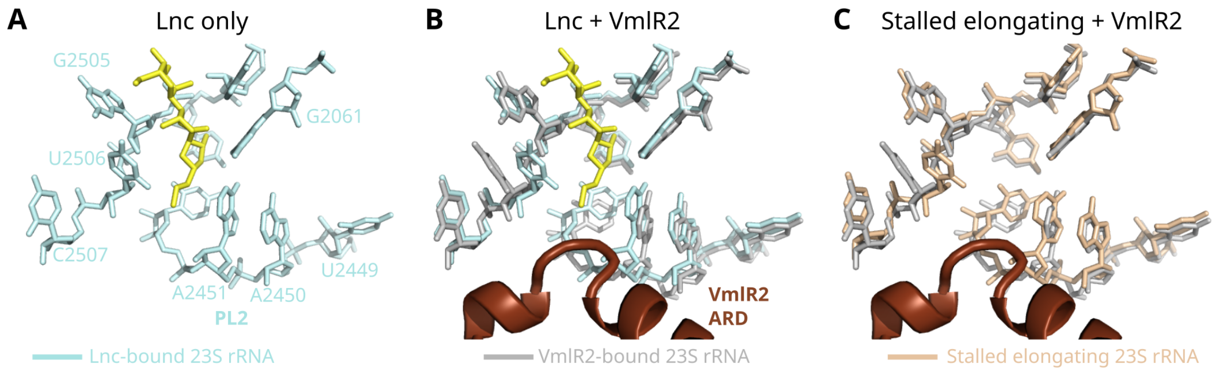

Supplementary Figure 11. VmlR2 modulates the conformation of 23S rRNA nucleotides around the PLS$_A$ binding site.

(**A**) View of lincomycin binding with selected 23S rRNA nucleotides shown in cyan (PDB ID 8A5I). (**B**) Same as **A** but with the VmlR2 structure superimposed. 23S rRNA from the VmlR2 model is shown in grey. (**C**) Same as B, except the VmlR2 structure is compared to a stalled elongating *B. subtilis* 70S ribosome (PDB ID 6HA1).

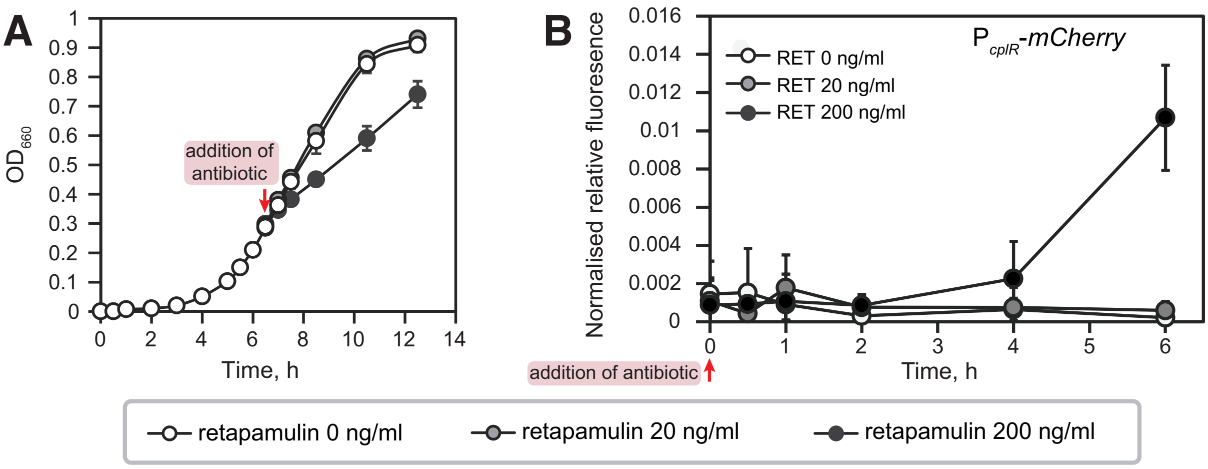

Supplementary Figure 12. Expression of P_{cplR}-*mCherry* fluorescent reporter is induced by retapamulin in a concentration-dependent manner.

(**A**) Growth kinetics of *C. difficile* in the absence or presence of sub-MIC concentrations of retapamulin (20 and 200 ng/ml). (**B**) Kinetics of the induction of the P_{cplR}-*mCherry* fluorescent reporter upon addition of retapamulin to final concentration of either 20 or 200 ng/ml. To calculate the relative promoter activity, relative fluorescence intensity normalised by $O.D._{600}$ was further normalised by the signal from the constitutive P_{fdx}-*mCherry* reporter.

Supplementary references:

1. Crowe-McAuliffe, C., Graf, M., Huter, P., Takada, H., Abdelshahid, M., Novacek, J., Murina, V., Atkinson, G.C., Hauryliuk, V. and Wilson, D.N. (2018) Structural basis for antibiotic resistance mediated by the *Bacillus subtilis* ABCF ATPase VmlR. *Proc Natl Acad Sci U S A*, **115**, 8978-8983.
2. Egorov, A.A. and Atkinson, G.C. (2022) uORF4u: a tool for annotation of conserved upstream open reading frames. *bioRxiv*, 2022.2010.2027.514069.
3. Polikanov, Y.S., Starosta, A.L., Juette, M.F., Altman, R.B., Terry, D.S., Lu, W., Burnett, B.J., Dinos, G., Reynolds, K.A., Blanchard, S.C. *et al.* (2015) Distinct tRNA Accommodation Intermediates Observed on the Ribosome with the Antibiotics Hygromycin A and A201A. *Mol Cell*, **58**, 832-844.
4. Schlunzen, F., Pyetan, E., Fucini, P., Yonath, A. and Harms, J.M. (2004) Inhibition of peptide bond formation by pleuromutilins: the structure of the 50S ribosomal subunit from Deinococcus radiodurans in complex with tiamulin. *Mol Microbiol*, **54**, 1287-1294.
5. Noeske, J., Huang, J., Olivier, N.B., Giacobbe, R.A., Zambrowski, M. and Cate, J.H. (2014) Synergy of streptogramin antibiotics occurs independently of their effects on translation. *Antimicrob Agents Chemother*, **58**, 5269-5279.
